# 

**Published:** 1828-04

**Authors:** 


### MONTHLY LIST OF MEDICAL BOOKS.

[*Medical Works cannot be entered on this List except a copy be sent for the purpose; the titles of Books having frequently been transmitted to us, as published, which have not appeared for weeks, or even months, after.*]

The Morbid Anatomy of the Brain. By ALEXANDER MONRO, M.D. F.R.S. & F.A.S.E. &c. &c. &c. Vol. I. Hydrocephalus. Illustrated by Copper-plates.—8vo. pp. 200. London, 1828.

A Practical and Pathological Inquiry into the Sources and Effects of Derangements of the Digestive Organs, embracing Dejection, and some other Affections of the Mind. By WM. COOKE, Esq.—8vo. pp. 290. London, 1828.

A Lecture upon Typhus Fever, as lately delivered at the Sunderland Infirmary. By WM. REID CLANNY, M.D. F.R.S.E. M.R.I.A.—4to. pp. 28.

A General View of the present State of Lunatics and Lunatic Asylums in Great Britain and Ireland, and in some other Kingdoms. By Sir ANDREW HALLIDAY, M.D. & K.H.—8vo. pp. 101. London, 1828.

Practical Observations on Insanity and the Treatment of the Insane; also Hints on the Propriety of making the Study of Mental Disorders a necessary adjunct to Medical Education.—8vo. pp. 127. London, 1828.

A Disquisition on the Nature and Properties of Living Animals. By GEORGE WARREN, Surgeon.—8vo. pp. 144. London, 1828.

Observations on Cow-Pox, and on the Necessity of adopting Legislative Measures for enforcing Vaccination. By H. EDMONDSTON, A.M.—8vo. pp. 156.

An Essay on the Diseases of the Jaws, and their Treatment. With two Plates. By LEONARD KOECKER, Surgeon Dentist.—8vo. pp. 95. 1828.

Cases illustrative of the immediate Effects of Acupuncturation, in Rheumatism, Lumbago, Sciatica, &c. By J. M. CHURCHILL, F.L.S. 8vo. pp. 101.

Oratio Harveiana Secunda. A ROBERTO BREE, M.D.—4to. pp. 28.

Proceedings at the Ninth Anniversary Meeting of the Hunterian Society, February 6th, 1828. With the Report, and List of Officers and Members.

---

### METEOROLOGICAL JOURNAL,

### NOTICES.

*Communications have been received from* Dr. BOW, E. G. B., Dr. LONG, Mr. HUMPAGE, *and* Mr. MACLEAN.

*We shall be happy to hear from* Dr. LONG *again.*

